# Effect of fluorine tin oxide substrate on the deposited SnO_2_: Ni thin films properties for gas sensing

**DOI:** 10.1016/j.heliyon.2024.e25585

**Published:** 2024-02-05

**Authors:** Khamael Ibrahim Abdulwahid, Chiheb Chaker, Hanen Chaker

**Affiliations:** a Department of Physics, College of Sciences, University of Misan, Maysan, Iraq; b Laboratory of Multifunctional Materials and Applications (LaMMA), LR16ES18, University of Sfax, Faculty of Sciences of Sfax, Sfax, Tunisia; c Laboratory of Materials and Environment for Sustainable Development (LR18ES10), University of Tunis El Manar, ISSBAT, Tunis, Tunisia

**Keywords:** Gas sensor, Ni-doped SnO_2_, FTO substrates

## Abstract

This study explores the deposition of Tin Oxide and Ni-doped SnO_2_ thin films (NSO) via spray pyrolysis from aqueous solutions. The deposition process was conducted under uniform conditions on two substrates, namely glass and fluorine tin oxide (FTO), with varying Ni percentages. The aim was to evaluate their potential for gas sensing applications. The as-deposited thin films exhibit diverse properties influenced by both Ni content and substrate type. X-Ray Diffraction (XRD) measurements reveal polycrystalline structures characterized by broad SnO_2_ diffraction lines, with the emergence of a NiO phase, particularly evident at higher Ni content. Notably, thin films deposited on FTO show the appearance of a secondary phase of SnO and enhanced crystallinity. Furthermore, lattice parameters and crystallite size decrease with increasing Ni percentage. The Field Emission Scanning Electron Microscopy (FE-SEM) analysis highlights significant alterations in surface nanostructures based on nickel content and substrate type. Higher nickel concentrations result in the formation of cauliflower-like structures, varying in size and density. This structural divergence significantly impacts the sensitivity of NSO-based NO_2_ gas sensors. Particularly, thin films with 20 % Ni, especially those deposited on FTO, exhibit optimal configurations for gas sensor applications, display sensitivity of 501 % at 100 ppm for nanocrystalline NSO/FTO compared to 436 % for glass-deposited samples. Our findings highlight the crucial role of Ni content and substrate type in modifying the structural and sensing properties of NSO thin films, for enhanced gas sensing applications.

## Highlights

• A low-cost chemo-resistance gas sensor with different proportions of Ni was synthesized by spray pyrolysis technique on glass, and FTO substrates.
• The XRD measurements show a nanocrystalline structure.
• The deposition on FTO substrate instead of glass substrates highly affects the structural and surface morphology.
• The sample resistance is highly sensitive to low concentrations of a target gas by the mechanism of adsorption-desorption of gas molecules.
• The optimum sample was the NSO with 20 % Ni deposited on FTO.

## 1 Introduction

Over the last decade, there has been a demand for the creation of extremely sensitive, low-temperature, compact, user-friendly, and stable gas sensors to monitor the environment for hazardous gases [1]. Metal oxides are among the most stable natural materials that can be used as sensitizers for a variety of hazardous gases, which are basic electronic circuits that can easily integrate [2]. Due to their advantages of being inexpensive, easy to build, and compact, metal oxide-based gas sensors are widely employed [3]. Due to their high stability, several metal oxides are generally used in gas sensors. SnO_2_ is the most commonly used material in gas sensing applications [4].

Tin oxide thin films (SnO_2_) have remarkable properties, making them suitable for various applications such as transparent conductive coatings in optoelectronic devices [5], solar cells [6] and gas sensors [7]. The undoped Tin oxide films exhibit excellent transparency and electrical conductivity [8], translation them crucial in developing technologies. Furthermore, the introduction of dopants into SnO_2_ thin films enhances their performance and extends their range of applications [9]. Doping with elements such as fluorine, indium, or antimony can further improve electrical conductivity, optical properties, and stability. This expand its use in advanced electronic devices, flexible electronics and energy storage systems [9]. Recently, there has been a high level of attention on the development of SnO_2_ nanostructures to enhance the performance of gas sensors [10].

Many studies are concentrated on pure and doped SnO_2_ for gas sensing due to its high stability, presence of native oxygen vacancies, and high charge carrier density [11]. Numerous studies have investigated various factors influencing the properties of SnO_2_ to achieve high-performance gas sensors, including elemental modification with many different and doped materials such as zinc [12], cobalt [13], etc., methods of preparation and nanostructure modification [14]. The composites usually exhibit improved performances compared to pure SnO_2_ in terms of gas sensitivity and response time. Nanostructure modification can be achieved by varying the substrate type and nature due to variations in the growth mechanism [15].

Bera et al. (2020) [16] synthesized oriented rutile SnO_2_ nanowires through atomic layer deposition seeding. Seeded growth controls the nucleation of nanowires and the crystallographic properties of seeds are key parameters for tuning the properties of nanowires. The prepared nanowires show efficient electrochemical CO_2_ reduction. Masuda (2020) [17] regulated the growth of SnO_2_ in aqueous solutions as nanosheets oriented along the (101) direction on FTO substrate for chemical sensors, without a seed layer. The (101) facet's broad, flat surface proved to be metastable. The constructed SnO_2_ nanosheet film had an approximate thickness of 800 nm and a gradient structure with several connections. By changing the etching condition, the metastable (101) facet can be used to influence the pace of crystal formation.

Our previous study (2023) [18] on SnO_2_ and NSO for gas sensing confirmed the tetragonal structure of pure SnO_2_ and NSO films. The lattice parameters decreased with an increasing Ni ratio. FE-SEM analysis revealed the emergence of cauliflower like aggregation structures with an approximate diameter of 100 nm attributed to the increasing Nickel content. Furthermore, these structures displayed a heightened density with higher percentage of Ni content. Raman spectroscopy indicates the formation of SnO_2_ nanostructures and an increase in defects and vacancies with increasing Ni contents. The NO_2_ gas sensors based on the NSO nanostructure showed enhanced performance toward NO_2_ at 100 °C, with the optimal Ni ratio of 20 mol %.

The objective of this study is to develop cost-effective, highly sensitive, low-temperature, and stable gas-sensing sensors for monitoring hazardous gases. The primary focus is on enhancing the performance of SnO_2_ thin film gas sensors through nanostructure modifications. The investigation encompasses the introduction of Ni dopants to enhance the properties of SnO_2_ thin films. Additionally, the study explores the impact of substrate type (glass or FTO) on the characteristics of deposited SnO_2_ thin films. The deposition technique employed is a simple one-step process using spray pyrolysis. A specific importance is placed on evaluating the applicability of these thin films in NO_2_ gas sensing applications.

## 2 Experimental

The flow chart for experimental work is shown below.

**Figure:**
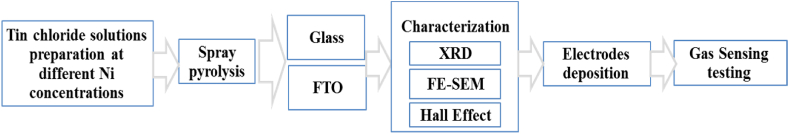

### 2.1 Synthesis

The starting materials that were used are: Sn Cl_2_.2H_2_O - 99.995°% purity, Merck Co. and NiCl_2_.6H_2_O - 99.9°% purity, Sigma-Aldrich. SnO_2_: Ni composite thin films were prepared at same deposition parameters on both glass and fluorine-tin oxide (FTO) substrates by spray pyrolysis from aqueous solution at 400 °C substrate temperature. Thin films were prepared at two Ni atomic ratios of 0.1 and 0.2. The atomizer was installed 30 cm above the substrates to spray the solution at 1.5 ml/min using 5 bars compressed air.

### 2.2 Characterization

The structural properties of SnO_2_ and NSO thin film were characterized using an X-Ray Diffraction system (Shimadzu XRD 6000) from 10 to 70° diffraction angle at 5°/min scanning speed. The surface morphology for the prepared samples was examined by FE-SEM (JSM-7600F by JEOL Ltd).The thicknesses of the thin films were measured using a reflectance probe (SR300 Å Sun Technologies). The dedicated software provided with the reflectometer device was utilized to determine the thickness, which was for all samples in the range of 310 ± 10 nm.

### 2.3 Fabrication of gas sensors

**Fig. 1:**
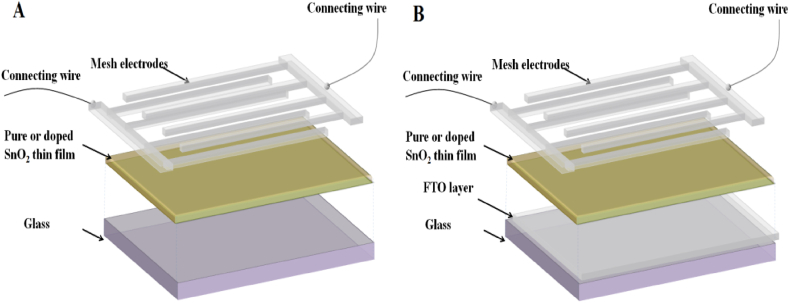
Schematic diagram for gas sensor structure on glass substrate (A) and on FTO (B).

Gas sensor devices were fabricated by depositing comb-like aluminum electrodes with a thickness of 200 nm onto the coating surfaces. For the samples deposited on glass and FTO substrates, a thermal evaporation technique was employed under a vacuum of 10^−5^ Torr using the Edwards coating system. The electrodes were connected by fine wires using silver paste. The schematic diagram of the gas sensor structure for the two configurations is illustrated in Fig. 1(A and B).

**Fig. 2:**
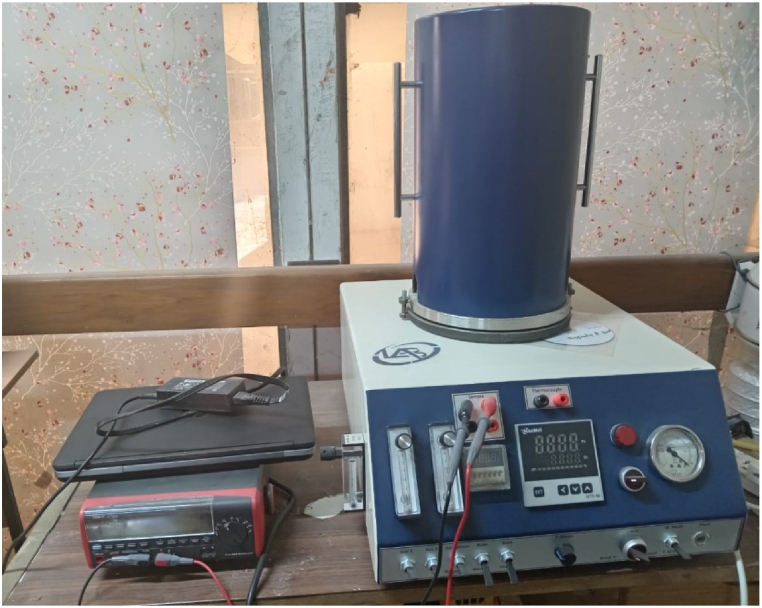
Image of the system for gas sensing testing.

Gas sensitivity for the SnO_2_ and NSO samples deposited on glass and FTO substrates were tested at a controlled temperature in a closed chamber vacuumed by a rotary pump. Electrically, the sample was connected through a multi-pin feed-through to a multimeter (Uni-T UT803 Benchtop Digital Multimeter) for resistance measurement connected to a computer during the examination. The Nitrogen dioxide gas (NO_2_) was mixed with air and flowed into the chamber through two flow meters and electric valves opened-closed for specific periods. Fig. 2 illustrates the image of testing system for gas sensing.

## 3 Results and discussions

### 3.1 Structural analysis

**Fig. 3:**
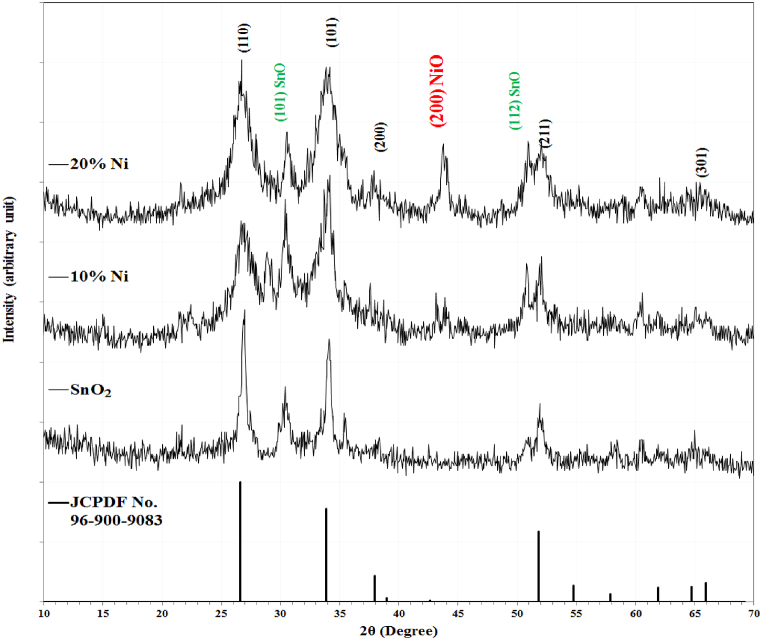
XRD for the SnO_2_ and NSO films at 10 and 20 % Ni ratios on FTO substrates.

The XRD patterns of as-deposited SnO_2_ and NSO composite thin films at 10 and 20 Ni at % on FTO substrates were shown in Fig. 3. A polycrystalline structure appeared for all samples. The dominant phase is the tetragonal SnO_2_, matched with the JCPDS Card No. 96-900-9083. Broad diffraction lines corresponding to (110), (101) and (211) for SnO_2_ were observed. The broad features of the diffraction lines indicated their nanostructure. The peaks are slightly shifted to higher diffraction angles with increasing NiO content, indicating the substitution doping of some Ni ions. Increasing the NiO ratio caused the presence of a separated NiO phase as a result of an increase in the Ni ion concentration beyond its solubility into the SnO_2_ lattice [19]. The samples deposited on the FTO exhibited higher crystallinity, and additional peaks corresponding to the SnO phase appeared according to JCPDS 96-901-2141, indicating the (101) and (112) planes at diffraction angles around 30° and 51°, respectively [20].

The following reactions (1) and (2) take place for SnCl_2_ in air:

(1) $${SnCl}_{2}+H_{2}O\to SnO+2HCl$$

(2) $$SnO+\frac{1}{2}O_{2}\to {SnO}_{2}$$

The reaction of SnCl_2_ forms intermediate molecules. Though Sn Cl_2_·2H_2_O can partly ionize into Sn^+2^ forming SnO structure.

Full width at half maximum increases with increasing Ni content, indicating a decrease in crystallinity, especially for thin films deposited on FTO substrates, suggesting a reduction in crystallite size. In general, replacing the glass substrate with FTO enhances crystalline growth, where the substrate of high crystallinity catalyzes the growth in a specific manner.

The separation distances between atomic planes (*d*_*hkl*_) were calculated from the angles of diffraction (θ) using Bragg's law (3) [21]:

(3) $$2d_{hkl}\;\sin\;\theta =n\lambda$$

where λ = 1.5406 Å is the used X-ray wavelength for Cu-K_α_, and *n* is the diffraction order. For the two types: on Glass and FTO substrates, the lattice parameters (*a* and *c*), for tetragonal SnO_2_ prepared at different Ni contents, were determined using equation (4) [21].

(4) $$\frac{1}{d_{hkl}^{2}}=\frac{h^{2}+k^{2}}{a^{2}}+\frac{l^{2}}{c^{2}}$$

While, using Scherrer formula [22], the crystallite size (*D*) was calculated with equation (5):

(5) $$D=\frac{0.94\;\lambda }{\beta \;\text{Cos}\theta }$$

Here, *β* represents the broadening of diffraction lines, determined through Lorentzian fitting using Match software.

While the micro-strain (ε) calculated according to the relation (6) [23]:

(6) $$\varepsilon =\frac{\beta }{4\;\tan\;\theta }$$

Table 1 listed the calculated lattice constants, crystallite size, and lattice strain for the SnO_2_ films with the content of Nickel on glass and FTO substrates. The crystallite size exhibits a decreasing trend with increasing Ni content, attributed to the substitutional doping with a lower radius ion, resulting in a reduction in lattice constants. Alongside, the lattice strain experiences an increment with higher Ni contents. Conversely, all samples deposited on glass substrates exhibit greater micro strain compared to those on FTO substrates, indicating a superior alignment of growth films on FTO due to its lower mismatch with the deposited lattice structure, as opposed to the randomly oriented atoms on the glass substrate.

**Table 1:** The lattice constants and the crystallite size (D) for the Ni–SnO_2_ on glass and on FTO.

NiO %	On glass				On FTO			
	a (Å)	c (Å)	D (nm)	ε × 10^−2^	a (Å)	c (Å)	D (nm)	ε × 10^−2^
**0**	4.73445	3.18204	8.2	1.92	4.76186	3.21788	15.2	1.04
**10**	4.70292	3.17320	7.5	2.10	4.74411	3.16997	9.5	1.66
**20**	4.69660	3.15660	6.4	2.46	4.73320	3.15808	8.6	1.83

### 3.2 Morphological analysis

**Fig. 4:**
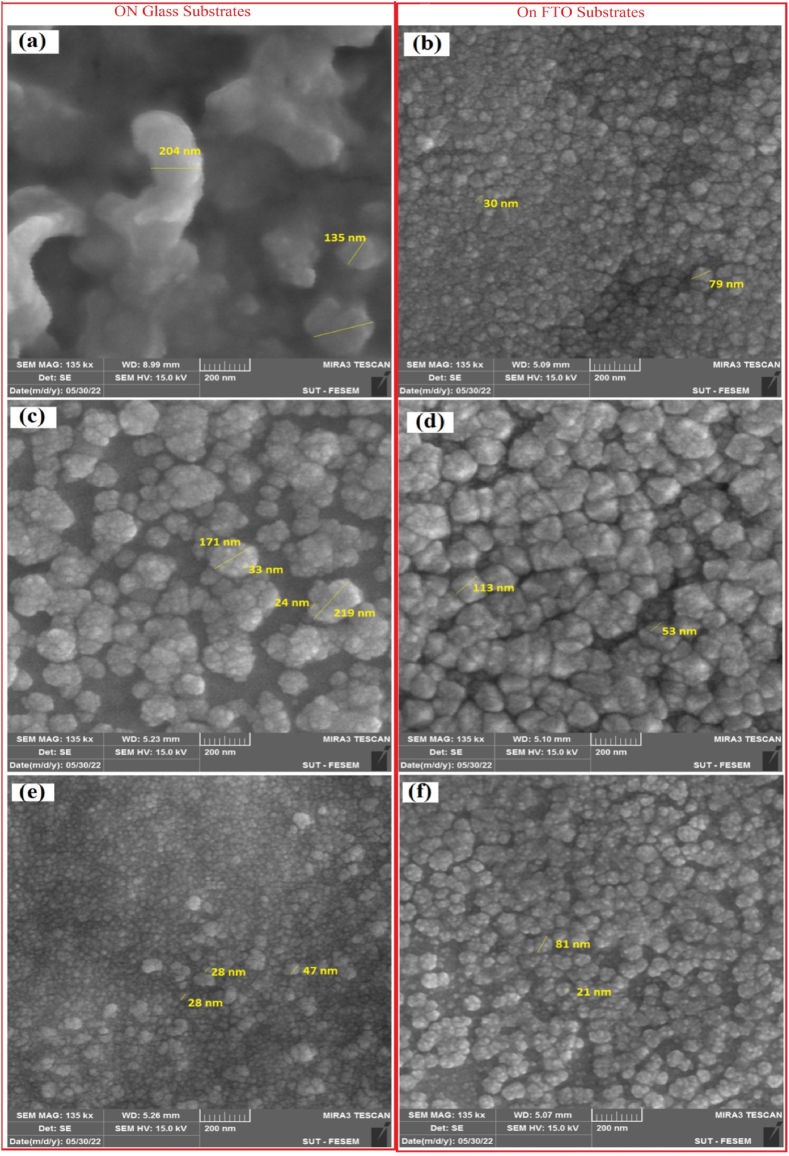
FE-SEM images for SnO_2_ on glass (a), SnO_2_ on FTO (b), NSO at 10 % Ni on glass (c), NSO at 10 % Ni on FTO (d), NSO at 20 % Ni on glass (e) and NSO at 20 % Ni on FTO (f).

The efficacy of gas sensing hinges significantly upon the surface morphology of the active layer, wherein the dimensions of nanostructures, porosity, and the inter-cluster connections' behavior play pivotal roles in gas sensing efficiency [24]. Illustrated in Fig. 4 are FE-SEM images portraying the as-deposited SnO_2_ and the NSO composite thin films with 10 % and 20 % Ni concentrations on both glass and FTO substrates. The samples deposited onto glass substrates (Fig. 4-a, c and e) exhibited irregular and non-uniformly distributed structures. The pure sample presented itself as a massive surface with small, irregularly dispersed fragments attached. In contrast, films deposited onto FTO substrates (Fig. 4-b, d and f) demonstrated thorough coverage of the substrate surface. Increasing the Ni content to 10 % induced the formation of cauliflower aggregation structures approximately 100 nm in diameter on the surface for both substrate types. These aggregations in the glass-deposited sample (Fig. 4-c) exhibited larger separations, while on FTO, they were denser and lacked any visible cracks or separations between adjacent nanostructures (Fig. 4-d). Subsequent increments in Ni concentration to 20 % led to a heightened density of these nanostructures, appearing interconnected without discernible separations. Consequently, samples deposited onto FTO substrates displayed a more regular pattern, with better-distributed nanostructures compared to those deposited onto glass. Additionally, the density of surface nanostructures increased with rising Ni concentration. Notably, the 10 % Ni concentration resulted in enhanced connectivity between structures, potentially facilitating improved mobility of charge carriers across grain boundaries and, consequently, enhancing efficiency in gas sensing applications [25]. The dopant type and substrate nature distinctly influence the microstructure governed by thin film nucleation and growth. Moreover, lattice disparities, particularly near the contact interface, contribute to increased stress in deposited films, affecting their physical properties, with thin films being more susceptible to such influences [26].

The measurement of Hall Effect was used to determine the electrical mobility, carrier concentration, conductivity and majority of charge carrier's types for the deposited SnO_2_ and NSO films at 10 and 20 % Ni ratios on FTO substrates as listed in Table 2. All films were n-type due to oxygen vacancies in metal oxide thin films. N-type semiconductors may play an important role in their electrical transport properties by forming an impurity level to release free electrons to the conduction band. The charge carrier mobility decreased from 6.77 to 2.40 cm^2^/Vs due to the creation of new grain boundaries against the charge carriers with the reducing the nanocrystalline structures, as shown in the FE-SEM test, with increasing the Ni percent from 0 to 20 %. The charge carrier concentration increased from 0.759 × 10^14^ to 1.517 × 10^14^ cm^−3^ for the same samples, which may be due to the formation of lattice defects by introducing the dopant, which is the source of charge carriers. The lowest mobility may enhance gas sensitivity, as shown by Li et al. [27], so the sample with 20 % Ni suggested has the best gas sensitivity. The final conductivity increased with the Ni dopant. This increment may be related to the lattice constant, which can be explained as follows: an increase in the lattice constant means that the electrons are less bound to the atom and can, therefore, be more easily removed, leading to a decrease in the band gap. Hence, the conductivity increases.

**Table 2:** Hall effect parameters for the SnO_2_ and NSO films at 10 and 20 % Ni ratios on FTO substrates.

NiO %	σ (Ωcm)^−1^x10^−5^	N_H_ cm^−3^ × 10^14^	μ_H_ cm^2^/Vs
**0**	5.82	0.759	6.77
**10**	6.76	1.093	3.87
**20**	8.23	1.517	2.40

### 3.3 Gas sensor measurements

**Fig. 5:**
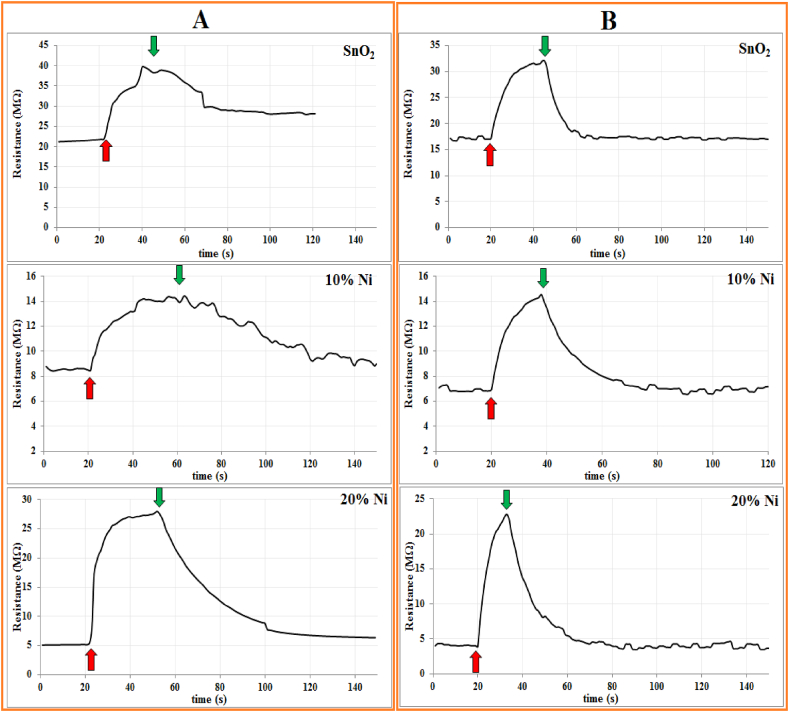
Resistance variation for the SnO_2_ and SnO_2_:Ni composite sensor at 100 °C operating temperature against 100 ppm NO_2_ on glass (A) and on FTO (B).

The gas sensor devices, fabricated on both glass and FTO substrates, underwent testing against NO_2_ gas. In Fig. 5(A and B), the resistance variation over time is illustrated at an operating temperature of 100 °C with 100 ppm NO_2_ for NSO/glass and NSO/FTO, featuring different Ni contents (0 %, 10 % and 20 %). In response to the reducing gas NO_2_, all samples demonstrated an increase in resistance. As anticipated, the samples exhibited n-type behavior attributed to the presence of oxygen vacancies, where the oxygen content falls below its stoichiometric ratio in the SnO_2_ structure [28].

Previously, ionized oxygen atoms and molecules from the atmosphere gained electrons from the conduction band of the sample, creating a depletion area whose effect depends on the size of the nanoparticles, which have a relatively large surface area. When oxidizing gas molecules pass, they interact with the surface atoms, leading to an increase in the thickness of the depletion layer. This, in turn, results in a decrease in the electrical conductivity of the sample relative to the gas concentration [25]. The decline in conductivity occurs through two primary mechanisms: a reduction in carrier concentration close to the surface of nanoparticles or a decrease in mobility across grain boundaries [24,29]. Specifically, in the matter of nanoparticles, the predominant impact stems from the alteration in mobility, attributed to the escalating barrier height across grain boundaries in tandem with an increase in depletion layer thickness [30].

Gas sensitivity was quantified using equation (7) [25]:

(7) $$S\%=\frac{R_{gas}-R_{air}}{R_{air}}\times 100\%\;\left[\text{Oxidizing}\;\text{gas}\right]$$

where *R*_*air*_ and *R*_*gas*_ represent the sensor resistance in clean-air and gas air mixture, respectively.

The sample blended with 20 % NiO demonstrated the highest sensitivity among all compounds. Across all samples, the recovery time consistently outpaced the response time, a characteristic attributed to the prolonged duration required for desorption reactions as listed in Table 3. The sensitivity exhibited a pronounced dependence on the nanoparticle structure's composition, with the 20 % Ni sample showing the highest sensitivity. Notably, samples deposited on FTO outperformed those on glass substrates. This superiority is attributed to the enhanced surface morphology of the FTO-deposited sample, where the grain boundaries play a pivotal role in modulating charge carriers by altering their potential barrier [31]. This nuanced variation in surface morphology contributes to the heightened sensitivity observed in this particular sample.

**Table 3:** Sensitivity, response time and recovery for the 20 % Ni-doped SnO_2_ sensor for sample deposited on glass and FTO against 100 ppm NO_2_ concentration.

Ni (%)	On glass			On FTO		
	Sensitivity (%)	Response time (s)	Recovery time (s)	Sensitivity (%)	Response time (s)	Recovery time (s)
**0**	79.1	18.0	25.0	89.0	22.0	24.0
**10**	62.8	25.0	60.0	112.3	20.0	40.0
**20**	436.0	10.0	45.0	501.4	17.6	36.0

**Fig. 6:**
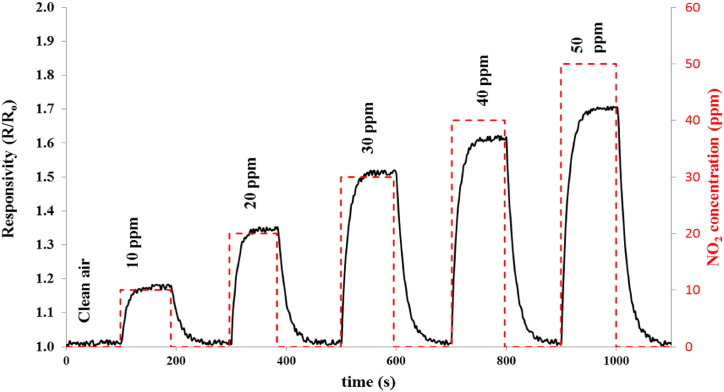
Responsivity variation at 100 °C for the 20 % Ni-doped SnO_2_ deposited on FTO against different NO_2_ concentrations.

**Fig. 7:**
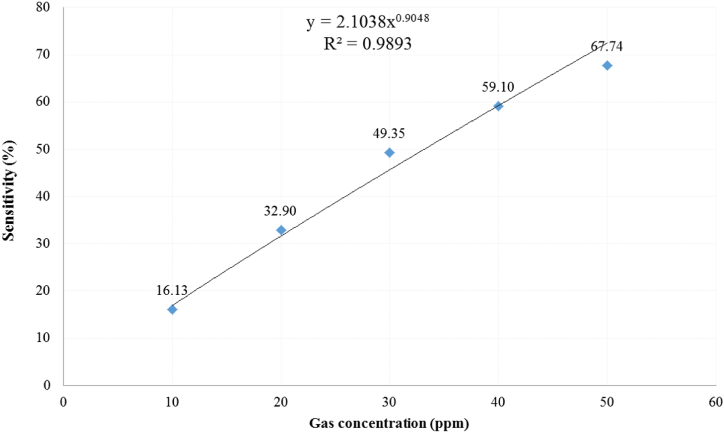
Sensitivity curve fit against the gas concentration at 100 °C for the 20 % Ni-doped SnO_2_ deposited on FTO.

The optimized sensor based on NSO at 20 % Ni on FTO was tested against NO_2_ gas at different concentrations from 10 to 50 ppm, at 100 °C operating temperature. Fig. 6 illustrates the variation of responsivity (the resistance in gas over the resistance in the air) with gas. The gas sensitivity trend increased exponentially (as the best fit) with gas concentration as shown in Fig. 7 according to the relations (8):

(8) S% = 2.1038 × (NO_2_ Con.[ppm])^0.9048^ [R^2^ = 0.9893]

**Fig. 8:**
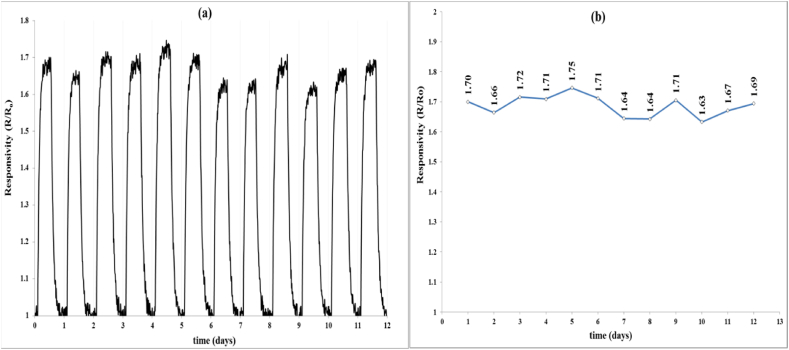
Long-term stability of Ni-doped SnO_2_/FTO sensor at 100 °C against 50 ppm NO_2_ gas: (a) time variation of gas response, and (b) maximum gas response.

Long-term stability is a crucial factor to sensors [32]. Fig. 8 (a,b) shows the long-term stability of the NSO/FTO sensor with same NO_2_ gas concentration of 50 ppm conducted at optimum operating temperature of 100 °C. It seems the high stability of the prepared sensor. The relative deviation of the gas response along 12 days is estimated to be 1.76 % and the average response value was calculated to be 1.68. The results indicate that the NSO/FTO sensor displays a good stability.

Table 4 illustrates a comparison between the specification of some previous gas sensors based on nanostructures of pure and doped SnO_2_ prepared by different techniques against different gasses with the current study. Although the sensitivity is lower than that previously reported, but the optimum working temperature at a lower temperature, in addition, operates at a wide range of detection than the compared studies.

**Table 4:** Comparison between the specification of previous SnO_2_-based gas sensors prepared by different techniques with the current study.

Sensor	Deposition technique	Target gas	Optimum operating Temp. (°C)	Detection range (ppm)	Sensitivity or response	Ref.
Ni-Doped SnO2	hydrothermal	HCHO	200	1–50	R = 68 at 50 ppm	[33]
Ni-Doped SnO_2_	Microwave-assisted wet chemical	CO_2_	275	10–200	S = 73 %	[34]
SnO_2_ nanofibers	Electrospinning	H_2_S	350	0.1–1	R = 15.2 at 1 ppm	[35]
SnO_2_ nanowires	Hydrothermal	H_2_	250	1–50	R = 13 at 40 ppm	[36]
Ni-doped SnO_2_ microstructures	Facile chemical solution	ethanol	260	1–100	R = 40 at 25 ppm	[37]
Ni- doped SnO_2_ thick films	Screen printing	LPG	300	600	S = 92 % at 600 ppm	[38]
Ni-doped SnO_2_ hollow spheres	Carbon microsphere template method	butanol	300	5–1500	R = 5.3 at 5 ppm	[39]
SnO_2_: In_2_O_3_	PLD	NO_2_	200	10–400	S = 300 % at 100 ppm	[40]
Nanocrystalline Ni- doped SnO_2_ thin films/glass	Spray pyrolysis	NO_2_	100	10–100	S = 436 % or R = 5.36 at 100 ppm	Current work
Nanocrystalline Ni- doped SnO_2_ thin films/FTO	Spray pyrolysis	NO_2_	100	10–100	S = 501 % or R = 6.01 at 100 ppm	

## 4 Conclusions

A low-cost chemo-resistance gas sensor based on NSO layers with different proportions of Ni was synthesized by spray pyrolysis technique on glass, and FTO substrates to investigate the effect of substrate structure on the deposited nano-film configuration and its effect on gas sensing performance. FE-SEM images test indicates the homogeneous NSO thin films with distinct nanostructure at a high Ni content. The XRD measurements show a nanocrystalline structure. The lattice parameters reduced with increasing the Ni compose ratio. The deposition on FTO substrate instead of glass substrates highly affects the structural and surface morphology. Concerning nanoparticles, a significant impact arises from the fluctuation in barrier height along the grain boundaries, influencing the behavior of charge carriers as the depletion layer thickness increases. Hence, the sample resistance is highly sensitive to low concentrations of a target gas by the mechanism of adsorption-desorption of gas molecules. The optimum sample was the NSO with 20 % Ni deposited on FTO. The proposed equation for sensitivity with gas concentration was achieved with a high R^2^ value. By comparing the results with those in previous research, despite the simplicity of manufacturing, a sensor was obtained that operates at a lower operating temperature compared to previous studies, with acceptable sensitivity specifications. Our findings not only contribute to the fundamental understanding of NSO thin films but also underscore the critical role played by Ni content and substrate type in modifying both structural and sensing properties. This study contributes in advancement of gas sensing applications through enhanced configurations of NSO thin films.

## Data availability statement

Data will be made available on request.

## CRediT authorship contribution statement

**Khamael Ibrahim Abdulwahid:** Writing – original draft. **Chiheb Chaker:** Supervision. **Hanen Chaker:** Formal analysis.

## Declaration of competing interest

The authors declare that they have no known competing financial interests or personal relationships that could have appeared to influence the work reported in this paper.
